# Editorial: Outbreak Investigation: Mental Health in the Times of Coronavirus (COVID-19)

**DOI:** 10.3389/fpsyt.2022.854388

**Published:** 2022-02-18

**Authors:** Ursula Werneke, Christina van Der Feltz-Cornelis, Bernd Löwe, Antonio Ventriglio, Dinesh Bhugra

**Affiliations:** ^1^Department of Clinical Sciences, Division of Psychiatry, Sunderby Research Unit, Umeå University, Umeå, Sweden; ^2^Department of Health Sciences, University of York, York, United Kingdom; ^3^Hull York Medical School, University of York, York, United Kingdom; ^4^Department of Psychosomatic Medicine and Psychotherapy, University Medical Center Hamburg-Eppendorf, Hamburg, Germany; ^5^Department of Clinical and Experimental Medicine University of Foggia, Foggia, Italy; ^6^Institute of Psychiatry, Psychologu & Neuroscience, Kings College London, London, United Kingdom

**Keywords:** COVID-19, coronavirus, pandemic, public health, mental health, psychological distress, anxiety, internet

Two years ago, on 20 January 2020, the World Health Organisation (WHO) declared the COVID-19 outbreak a public health emergency of international concern. At that time, it was unclear what lay ahead in terms of its impact on mental health of populations, although some lessons from previous epidemics and pandemics were already available. With all focus on physical survival, mental health was put on the back burner. However, concerns started building up rapidly. How would people without a history of mental health problems cope with the psychological fall-out from the COVID-19 pandemic? And how would those with already existing mental health problems cope? To monitor this unfolding mental health crisis, we embarked on our Research Topic on 3 April 2020. At that time, there were over one million COVID-19 cases recorded worldwide. The death toll had surpassed 50,000. Two years later, at the time of writing this editorial on 13 January 2022, there were over 300 million cases recorded world-wide. The death toll had surpassed 5.5 million people (1).

The recent Omicron variant has made it clear that COVID-19 is not going away. But this may not necessarily mean doom. The Omicron variant, albeit much more transmissible, seems much milder. Cases have surged globally, hospitalization and death rates have not. Preprint data from South Africa, where the Omicron variant was first reported, indicates 80% reduced odds for hospitalization and 70% reduced odds for severe disease (2). Still, due to the sheer numbers of infections, the death toll may rise. Concerns about hospital and intensive care capacities have led some countries to re-introduce lock-down measures. This has caused havoc in vulnerable industries, such as tourism and gastronomy. They had their hopes set on a strong rebound during the 2021 festive season, only to shut down again. However, even that situation is changing rapidly. The milder course of Omicron and a shorter incubation time [3 days (3)] suggest that self-isolation measures could be relaxed. On 27 December 2021, the US Centers for Disease Control and Prevention (CDC) shortened quarantine time from 10 to 5 days for people testing positive but asymptomatic (4). South Africa went even further, scrapping the need for isolation altogether in asymptomatic individuals. One day later though, after an intense flood of media, stakeholders and public enquiries, the new isolation and quarantine rules were recalled (5). Meanwhile, Germany discusses school closures (6).

The most predictable thing about the pandemic in the new year is its utter unpredictability. Three factors may become game changers in 2022: vaccines, anti-COVID medicines and economic performance. Globally, more than 9 billion vaccine doses have been set (7), however, COVID-19 vaccine coverage remains patchy. In richer countries, anti-vax influencers, including conspiracy theorists, populists, and some vocal celebrities, continue to undermine the confidence in the vaccines, driving down vaccination rates. In poorer countries, vaccine supplies continue to be limited. Incomplete vaccination coverage, though, increases the likelihood of further mutations. Vaccine developers may not be able to keep up with the speed of mutations, unless they manage to predict mutations before they occur and decide to mass-produce vaccines based on their predictions. As with Omicron, future COVID-19 strains may turn out more transmissible but less virulent than previous ones. But this may not invariably be the case. For instance, a future mutation could cause the virus to replicate in people's airways at higher levels than the immune system could clear. This would also lengthen the time an infection persists (8). Antiviral medicines such as Paxlovid (nirmatrelvir tablets and ritonavir tablets, co-packaged for oral use) and Lagevrio (molnupiravir) may become the next movers in the equation. Approved for emergency use by the US Federal Drug Administration (FDA) on 22 and 23 December 2021 (9, 10), both medicines can be taken in the convenience of one's home. However, the tablets are not a substitute for vaccination (9). These anti-COVID medicines are expensive and most likely out of reach for many countries. Poorer countries may acquire supplies through donations and subsidies, often *ad hoc* and one-off. Under such circumstances, securing steady supplies may be economically unviable. Finally, how well the world fares in terms of mental health will depend on economic performance as well. Fiscal stimulus packages have been one way of taking people through the economic fallout of the pandemic. But fiscal stimuli together with labor and supply shortages have driven up inflation (11). Soaring energy prices add further inflationary pressure. Compensatory rises of interest rates may put pressure on indebted countries and households alike. The longer the pandemics persists, the more economies will become vulnerable. It is indeed difficult to predict where the world is heading in 2022. Much of the uncertainty we have experienced since the beginning of the pandemic will prevail. Other uncertainties will emerge. Uncertainty makes people anxious. Uncertainty that does not go away makes people depressed.

Mental health impact of pandemic can be seen as occurring due to anxiety, loneliness, and isolation but also grief due to loss of friends and family without likelihood of being able to attend funerals in early days and survivor guilt. Furthermore, long COVID brings with it certain mental health factors into play. Understanding and documenting the early impact of the COVID-19 pandemic on mental health can help us to manage the challenges lying ahead. Early experiences, frozen in time, provide a unique historical account of the unfolding pandemic. This Research Topic is an investigation into the early impact of the COVID-19 pandemic on mental health. It has covered nearly all aspects of mental health during the pandemic. Most of the studies came from China and Italy, two countries particularly hard-hit in the early days of the pandemic. Many of the studies published here were based on online surveys. The discussions of their strengths and limitations leave a vivid testimony of epidemiological research during the lockdown, replacing fieldwork on the ground with fieldwork in cyberspace.

Some findings were expected. Undoubtedly, the pandemic has precipitated psychological distress, trauma, anxiety and affected quality of life as depicted in several contributions in this collection (Akdeniz et al.; Bottaccioli et al.; Chen et al.; Cerami et al.; Ferreira dos Santos et al.; Ganesan et al.; Luo et al.; López Steinmetz et al.; Rondung et al.; Yan et al.). One study from Pakistan, however, did not find any increase of anxiety and obsession in the surveyed population. The authors speculated that might either be due to resilience or lack of understanding (Majeed et al.). It is also possible that in cultures where fatalism is a common response, people may deal with it in a different way. More research is on its way concerning the psychological impact of COVID-19 (Giallornardo et al.; Schimmenti et al.).

The evidence collated in this Research Topic also points toward a level of gender disparity. Men run a higher risk of an adverse clinical course and death of COVID-19 infection (12). But women seem to bear the psychological brunt of the pandemic (Dagnino et al.; Parlapani et al.; Rossi et al.; Thomas et al.; Torales et al.). Admittedly, women are more likely to participate in surveys. Thus, some selection bias may have been at play. Yet, this gender difference is not entirely unexpected. In many parts of the world, women carry a higher burden of caring for families. They tend to be more isolated and run a higher risk of economic hardship and insecure employment. Social and geographical isolation may promote intimate partner violence (Mojahed et al.). Alcohol use may rise during traumatic events, global disasters, and economic crisis, in part mediated by anxiety, depression and post-traumatic stress. Here, being male, young, or single may convey the highest risk (Gonçalves et al.). However, addiction was only covered in one contribution to this Research Topic, exploring gambling activity in Sweden. Rather unexpectedly, gambling activity declined during the first phase of the outbreak compared to a previous forecast (Lindner et al.).

Health care workers were another group of individuals identified to experience high levels of psychological distress at the beginning of the pandemic (Cao et al.; Liu et al.; Nguyen et al.; Zhang, Xie, et al.; Zhang, Wang, et al.). Unsurprisingly frontline medical staff may be most vulnerable (Zhang, Zhao, et al.). Professional self-identity, good psychological preparation, social support, and positive cognitions were protective (Mo et al.; Xie et al.). Two contributions gave examples of how support systems for hospital staff could work in practice (Geoffroy et al.; Rolling et al.). At the same time access to protection equipment including face masks proved essential to maintain psychological health (Lam et al.). The scarcity of protective equipment at the beginning of the pandemic is a testimony of unpreparedness of authorities all over the world for major disaster. For the individual health care worker, forced to work without protective equipment, this may have led to moral injury and subsequent post-traumatic stress. Moral injury has mainly been examined in the context of warfare and military service. It can present when there has been a betrayal of what is right, by someone who holds a legitimate authority, in a high-stake situation (13). But the beginnings of the pandemic did not only leave health care workers highly vulnerable. Patients, particularly when suffering from severe conditions such as cancer, may have been at an increased risk of post-traumatic stress, when resources were withdrawn and treatments were delayed (Bandinelli et al.; Cui et al.; Li et al.).

The beginnings of the pandemic may have been particularly difficult for people who already had mental health problems. As the focus on somatic health eclipsed mental health needs, people with mental health problems became less visible. Two studies in this Research Topic, describing a decreased use of psychiatric inpatient and emergency services in adults and adolescents, give testimony to this effect (Kim et al.; Díaz de Neira et al.). Patients, however, may have struggled in silence at home, as demonstrated by a interview studies of patients with eating disorders and obsessive compulsive disorders (McCombie et al.; Benatti et al.). Individuals with pre-existing mental health problems may also have run into more physical problems during the pandemic. A Swedish register study showed that the odds of COVID-19 associated death was double in people with psychotic or bipolar disorder (Maripuu et al.). Further work has shown that this increased mortality may not be specific to COVID-19; similarly increased odds arise with other lung infections (14). As judged by increased hospitalization rates (15), the higher mortality risk may be more likely linked to an increased risk of an adverse clinical course of a COVID-19 infection than to an increased risk of infection *per se*. Efforts to prioritize people with serious mental disorder for vaccination must continue.

Several observational studies in the Research Topic explored coping strategies. Positive thinking and reducing cognitive bias may be strategies worth trying (Baldacara et al.; Giusti et al.; Shudy et al.). Religiosity may also be protective (Saleem et al.). Physical exercise may reduce stress levels and build resilience (López-Bueno et al.; Bento Silva et al.; Van Der Feltz Cornelis et al.). Face masks seem to offer protection against COVID-19 infection, even if robust randomized controlled studies are still lacking (16, 17). Intriguingly, face masks may also have an impact on mental health. One study comparing the impact of face mask use in two countries showed that use of face mask was associated with less anxiety, depression, and stress (Wang et al.). Possibly, taking control by using a mask reduces feelings of stress, anxiety, and depression. Although this finding can at best be considered preliminary, it is still noteworthy. Finally, right from the beginning of the pandemic, there was a proliferation of COVID-19 related health apps, providing news and information, contact tracing, and self-assessment, or diagnosis (Zhang, Chow, et al.). Such apps may facilitate infection control and help to stay connected in periods of quarantine. However, there are caveats to the current “infodemic” (18). Civil liberties may become infringed when there is comprehensive control of movements. At the same time, a constant flow of information may increase stress, particularly in people who are intolerant to uncertainty. Besides, not all apps are equally reliable. They may be used to spread misinformation and conspiracy theories. The verdict is still out whether such apps do more harm than good. Virtual reality applications generating positive emotions to may take self-help to the next frontier. A randomized trial is planned to test whether such a virtual reality protocol can be used to improve wellbeing and preserve social connectedness through the beneficial social effects (Riva et al.).

The economic impact of the COVID-19 pandemic may play as much of an essential role as the infection itself in short, medium, and long-term. Strict lockdowns, implemented almost globally, have brought economic insecurity and poverty for many. Job and income loss, as well as the fear of it, add to anxiety and depression. A statistical modeling exercise based on longitudinal data from 38 countries showed that unemployment might increase suicide rates, particularly in middle-age. Loss of national income might even have a higher impact on suicide rates, particularly in the older age groups (Brenner and Bhugra). Currently, in many high-income countries, vaccination programmes are being implemented at an exponential rate. At the same time, our ability to effectively treat severe courses of COVID-19 infection has substantially improved. The subsequent reduction of mortality will invariably shift the focus to economic and social recovery, even if new outbreak waves and mutations lie ahead. Such may then precipitate further mental health problems in an already primed population. At present, it remains unclear whether the detrimental effect of COVID-19 on mental health is transitory or lasting. A global study estimated an additional 53.2 million cases of depression and an additional 76.2 million cases of anxiety due to COVID-19 for 2020 (19). But depending on data sources and circumstances, there is scope for over-reporting and observation bias. For instance, according to that study, Sweden should have experienced a 22–25% change in the prevalence of major depression after adjustment for COVID-19 (19). Data extrapolated from the Swedish Board for Health and Welfare suggest otherwise. Depression requiring specialist services and suicide rates have not gone up in 2020 (20). Prescribing for antidepressants has increased by little more than one percent (21). This is consistent with suicide trends observed in 21 countries or areas within a country during the beginning of the pandemic. Suicide numbers remained largely unchanged or even decreased in same countries or areas (22). However, it remains unclear whether these findings from high- and upper-middle-income countries can be generalized to lower-middle- and low-income countries.

The pandemic is not over yet. COVID-19 related mental health problems and their consequences are likely to be with us for a long time. Neither is the direct impact of long-term COVID-19 on mental health fully understood. Some survivors of COVID-19 are even at risk of psychiatric sequelae, which may either be caused by the virus itself or the immune response to it (23). Ultimately, it is early days. And it may not take another 100 years until the next pandemic. We hope that the lessons learnt in these early days of the COVID-19 pandemic and documented in this Research Topic can be used in preparation for the next one.

## Author Contributions

UW initially proposed and set up this Research Topic and led the editorial introduction. All authors acted as guest editors, managed the submissions, and worked collaboratively to decide which manuscripts were accepted or rejected. All authors contributed to the article and approved the submitted version.

## Conflict of Interest

UW has received funding for educational activities on behalf of Norrbotten Region (Masterclass Psychiatry Programme 2014–2018 and EAPM 2016, Luleå, Sweden) from Astra Zeneca, Eli Lilly, Janssen, Novartis, Otsuka/Lundbeck, Servier, Shire, and Sunovion, and lecture honoraria from Lundbeck. DB has lectured for Lundbeck Foundation and Janssen in the past. The remaining authors declare that the research was conducted in the absence of any commercial or financial relationships that could be construed as a potential conflict of interest.

## Publisher's Note

All claims expressed in this article are solely those of the authors and do not necessarily represent those of their affiliated organizations, or those of the publisher, the editors and the reviewers. Any product that may be evaluated in this article, or claim that may be made by its manufacturer, is not guaranteed or endorsed by the publisher.
